# Collagen-Sealed Polyester Vascular Prostheses Functionalized by Polycatecholamine Coatings

**DOI:** 10.3390/ijms23169369

**Published:** 2022-08-19

**Authors:** Anna Michalicha, Cristina Canal, Albert Espona-Noguera, Mateusz Piet, Barbara Budzyńska, Stanislaw Przywara, Anna Belcarz

**Affiliations:** 1 Chair and Department of Biochemistry and Biotechnology, Medical University of Lublin, Chodzki 1, 20-093 Lublin, Poland; 2 Biomaterials, Biomechanics and Tissue Engineering Group, Materials Science and Engineering Department, and Research Center for Biomedical Engineering, Technical University of Catalonia (UPC), Escola d’Enginyeria Barcelona Est (EEBE), C/Eduard Maristany 14, 08019 Barcelona, Spain; 3 Barcelona Research Center in Multiscale Science and Engineering, UPC, 08019 Barcelona, Spain; 4 Department of Virology and Immunology, Maria Curie-Sklodowska University, Akademicka Street 19, 20-033 Lublin, Poland; 5 Independent Laboratory of Behavioral Studies, Medical University of Lublin, Chodzki 4a, 20-093 Lublin, Poland; 6 Department of Vascular Surgery and Angiology, Medical University of Lublin, Staszica 11, 20-081 Lublin, Poland

**Keywords:** collagen-sealed vascular prostheses, poly(L-DOPA), gentamicin, glycerol, antibacterial

## Abstract

Collagen-sealed polyester (PET) prostheses are commonly used in reconstructive vascular surgery due to their self-sealing properties. To prevent post-surgical infection, different modification methods have been tested but so far none have showed long-term satisfactory efficiency. For this reason, in the present study, a commercial collagen-sealed PET prosthesis was coated by a highly adhesive poly (L-DOPA) layer maintaining the sealing protein without losing the original properties and functionality. This modified (as proven by SEM, FTIR, XPS and contact angle) graft exhibited comparable wettability and elasticity as pristine commercial graft, as well as reduced hemolysis-inducing effect, lowered toxicity against human endothelial cells and reduced toxicity in Danio rerio model. Poly (L-DOPA)-coated grafts were shown to bind six times more aminoglycoside antibiotic (gentamicin) than pristine graft. Poly (L-DOPA)-coated antibiotic-bound prostheses exhibited an improved antibacterial activity (bacterial growth inhibition and anti-adhesive capacity) in comparison with pristine antibiotic-bound graft. Overall, poly (L-DOPA)-coatings deposited on PET vascular grafts can effectively functionalize collagen-sealed prostheses without the loss of protein sealing layer and allow for antibiotics incorporation to provide higher safety in biomedical applications.

## 1. Introduction

Polyester (PET) vascular prostheses, usually named Dacron^R^ grafts, are nowadays widely used in reconstructive vascular surgery. Over the period of 60 years, since their first implantations in humans, in 50th ties of the last century [1], the polyester grafts have undergone multiple biochemical modifications to make them more and more safe and effective for applications in vascular patients. One of the most crucial modifications was related to blood sealing properties of PET tubes, which resulted in albumin- or collagen-sealed PET grafts [2,3]. This specific modification is now present in the majority of PET vascular grafts available on the market, with prevalence of the collagen coating, which has been found to be optimal. Impregnation with collagen makes the PET prosthesis non-permeable for blood and thus ready for implantation without any previous preparation procedures as so-called pre-clotting of unsealed PET prostheses. Collagen is not only compatible with synthetic polymers but also non-toxic, hemostatic, biodegradable and highly susceptible to various modifications. This last feature allows one to control the collagen degradation rate (via cross-linking with formaldehyde, glutaraldehyde, carbodiimide, acylazide, examethylenediisocyanate etc.) and offers a platform to synthesize a variety of collagen-based systems for drug delivery (including vancomycin, trimethoprim, amphotericin-B, gentamycin, polymyxin-B sulfate and tobramycin) [4,5,6]. This made collagen coating a milestone in reconstructive vascular surgery.

However, the problem of early or late infection of vascular prostheses still remains the Achilles’ heel of modern vascular surgery. Vascular graft infection occurs in 0.6 to 5% of patients after reconstructions in aorto-iliac level. Mortality in this group is estimated to be between 25–88% [7]. Moreover, 40–70% of the patients will undergo major amputation [8]. To reduce this problem, silver- and rifampicin-bounded grafts were produced and they became the most frequently used collagen-coated grafts. However, they are definitely not the final solution for this serious and possibly lethal outcome complication [9]. The rate of re-infections of silver coated grafts in the femoral-popliteal segment is reported to be 19% [10]. Continuity of infection and bacterial resistance was found in 31% of patients after replacement of infected vascular graft with rifampicin coated prostheses [11]. Therefore, there is still a strong clinical need for further attempts at biochemical modification of PET vascular prostheses in order to create a product which could be maximally resistant to early or late vascular graft infection.

In the quest for the solution to this problem, one should remember that collagen must be treated with care to retain its natural properties. Its native triple helix conformation is maintained at low temperatures (30–40 °C) [12], and strong alkaline pH may cause its disturbance in restoration of thick fibril-bundle structures [13] while acidic pH may cause collagen dissolution. Therefore, mild collagen modification conditions must be selected to bind antibacterial factors. In 2007, a new method to deposit a multifunctional polydopamine polymer on various substrates through simple dip-coating in aqueous dopamine (compound of catecholamine family) solution was reported [14]. Since then, the described method has attracted growing attention, not only due to the feasibility of coating deposition but also to its multifunctionality [15]. Among others, polydopamine (PDA) can be a platform (due to the presence of catechol moieties) for attachment of antibacterial or antifouling agents containing free amino and thiol groups. Moreover, it allows for the attachment and encapsulation of living cells, serves as “primer” for in situ silver nanoparticle deposition, increases the surface hydrophilicity and decreases the toxicity of coated substrates [16]. Polydopamine coating also increases the attachment and spreading of human endothelial cells on polycaprolactone fibers and stainless steel [17,18]. Interestingly, not only dopamine but also its biochemical precursor levodopa (L-DOPA) can form coatings on different substrates. L-DOPA was already reported as more beneficial (in terms of modification of polyester fibers with antibiotics) than dopamine [19]. We have recently reported the deposition of polymerized L-DOPA (PLD) on natural curdlan (β-1,3-glucan) and synthetic polyester fibers (of complex 3D structure), followed by gentamicin immobilization on the coatings. Both curdlan and polyester fibers, coated with PLD, exhibited higher antibiotic binding yield and antimicrobial activity in comparison with the same fibers coated with PDA [19,20,21]. It was also shown that human endothelial cells proliferated better on PLD-coated polyester fibers than on the pristine ones and that this modification reduced the toxicity against Danio rerio embryos [19].

To our knowledge, a strategy for polycatecholamine-based functionalization of collagen-sealed vascular prostheses has never been proposed before. Therefore, in this study we tested the possibility of polycatecholamine grafting on collagen-sealed polyester vascular prostheses. In a pilot experiment, we tested both PDA (polydopamine) and PLD (polymerized L-DOPA) as coatings and then we selected the most promising one. First, we wanted to prove that coating of collagen-sealed vascular grafts using polycatecholamines is possible and that the procedure is effective without the loss of the collagen-sealing layer and important properties of the grafts (their hydrophilicity, elasticity, favored attachment and proliferation of endothelial cells and biological safety). Second, we hypothesized that the grafted polycatecholamine layer can serve as a platform for further immobilization of antibiotics to protect the collagen-sealed grafts against bacterial adhesion and growth.

## 2. Results and Discussion

In our previous work, the coating with polycatecholamine layers was performed on polyester knitted vascular grafts [19]. Despite their complex shape, the coating was relatively uniform and it enabled the immobilization of significant amounts of gentamicin via catechol moieties of polycatecholamines (PDA produced from dopamine and PLD produced from L-DOPA as monomers, respectively) [19]. Thus, we decided to test similar coating conditions for modification of collagen-sealed prostheses, although relative sensitivity of collagen fibers to chemical environment was a challenging factor. According to the manufacturer, the graft selected for our study was sealed with bovine dehydrothermal crosslinked collagen type I mixed with glycerol before the graft coating.

Overall, optimization of the process of collagen-sealed graft coating with polycatecholamines, as demonstrated in Supporting Information, showed that the most promising results were obtained when the following conditions were used: 10 mM Tris buffer pH 8.5, addition of Cu^2+^, Na^+^ and Mg^2+^ ions, coating temperature 37 °C and L-DOPA as a monomer (which polymerized into PLD coating). This set of conditions allowed us to produce the grafts with a uniform (according to visual observations) PLD layer which enabled binding maximum gentamicin amount with the highest antibacterial activity. Therefore, this set of conditions was used for prostheses coating in all further experiments. However, during the pilot experiments we observed that the coating procedure caused the decrease of dry prosthesis weight (by approximately 50%) and significant increase of stiffness and brittleness. This second phenomenon is particularly undesirable for vascular grafts because it hinders the procedure of graft implantation and suturing with the blood vessel wall. We found that glycerol removal during the coating process is responsible for this phenomenon. Thus, we performed a series of tests aiming to restore the grafts’ flexibility by reintroduction of glycerol into their structure (data not shown). We found that simple soaking of PLD-coated grafts in 30% aqueous glycerol solution at 25 °C for 10 min, followed by drying at 37 °C, restored the flexibility and initial weight of the prostheses. Thus, PLD-coated glycerol-regenerated grafts were also subjected to further tests. Table 1 describes the samples used in these experiments.

**Figure 1:**
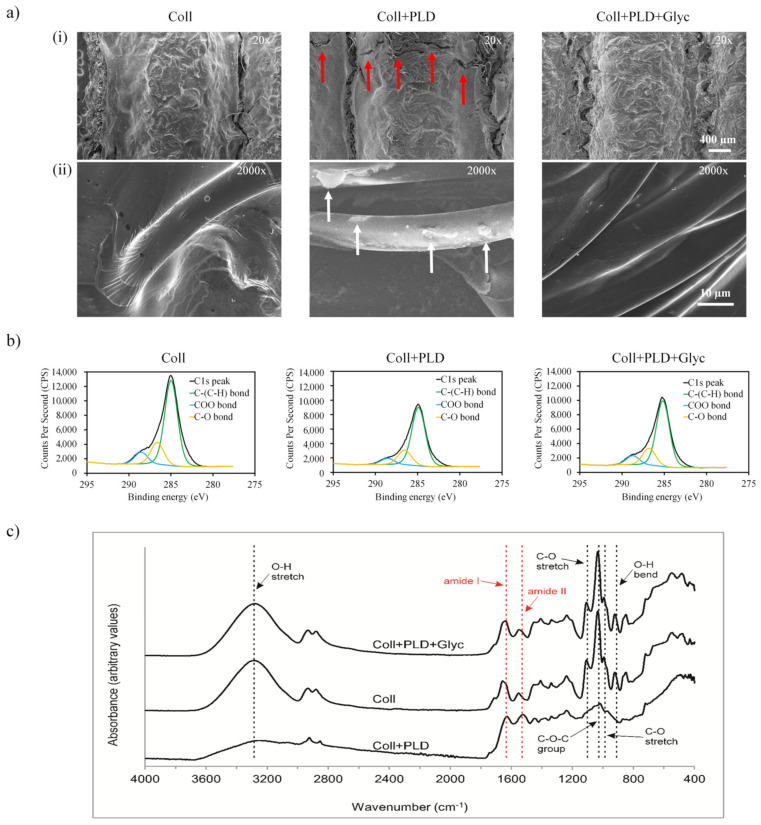
(**a**) SEM micrographs of Coll, Coll+PLD and Coll+PLD+Glyc vascular grafts at (**i**) 20× magnification and (**ii**) 2000× magnification. Scale bars: 400 μm and 10 μm. (**b**) XPS C1s peak deconvolution showing the peaks corresponding to C–(C/H), C–O and COO bonds of Coll, Coll+PLD and Coll+PLD+Glyc samples. (**c**) ATR-FTIR spectrum of Coll, Coll+PLD and Coll+PLD+Glyc grafts. Glycerol-related bands were marked in black and collagen-related bands were marked in red.

The first hypothesis put forward in our study was that our optimized coating procedure did not disrupt the collagen-sealing layer and left intact the most important properties of the grafts, such as surface topography and chemical composition, hydrophilicity, elasticity, favored attachment and proliferation of endothelial cells and biological safety. Therefore, PLD-coated grafts (both glycerol-regenerated and non-regenerated) were subjected to series of experiments and compared with pristine grafts. In the first place, we evaluated the surface topography of Coll, Coll+PLD and Coll+PLD+Glyc samples by SEM (Figure 1a). All the tubular knitted samples showed similar macrostructure showing a thick layer of coating over the polyester multifilaments. However, some microcracks were observed in the Coll+PLD vascular grafts (indicated by red arrows in Figure 1(ai)) which were not present in the Coll and Coll+PLD+Glyc samples. At higher magnification (2000×), the surface of the fibers of Coll and Coll+PLD+Glyc samples were similar, both showing a smooth topography (Figure 1(aii)), with a polymeric coating bonding the fibers. In contrast, the surface of Coll+PLD vascular grafts displayed the individual fibers without the continuous coating, which was only observed as rough irregular deposits on the surface of the fibers (indicated by white arrows).

In order to further characterize the studied samples, the chemical nature of the different surfaces was evaluated by XPS and ATR-FTIR analysis. The chemical composition of the first nanometers of the surface revealed the presence of C1s, O1s and N1s, in agreement with the structure of collagen (Table 2). Following PLD coating, the O/C ratio and N/C decreased in Coll+PLD samples reflecting a higher C amount in the PLD molecules than in collagen and, thus, the presence of the molecule on the surface—from 0.2039 (Coll) to 0.1201 (Coll+PLD) in O/C and from 0.0670 (Coll) to 0.0458 (Coll+PLD) in N/C. After the glycerol soaking process, Coll+PLD+Glyc vascular grafts displayed similar values to the Coll sample, which might indicate that glycerol is not retained on the surface but rather mainly absorbed by the graft structure. To determine the percentage of the different C bonds present in the surface of the studied vascular grafts, the C1s peak deconvolution reflected the presence of C-(C/H), C-O or C-N and COO bonds (Figure 1b). In all the samples, C-(C/H) bond was the most predominant C bond, followed by C-O and COO. Comparing Coll and Coll+PLD samples, the PLD coating caused an increase of C-(C/H) from 71.43% to 73.48%, while C-O bonds remained similar (18.54% for Coll and 18.67 for Coll+PLD) and COO bonds decreased from 10.04% to 7.86%. Then, after the glycerol soaking process, Coll+PLD+Glyc vascular grafts showed similar values to the Coll samples, with 71.55% for C-(C/H), 18.18% C-O and 10.26% for COO. This phenomenon may be a consequence of glycerol contamination with fatty acids which contain carboxyl groups in their chain (glycerol is frequently produced by transesterification of fatty acids) [22].

The ATR-FTIR spectral analysis of Coll, Coll+PLD and Coll+PLD+Glyc samples (Figure 1c) confirmed the presence of amide I (1640 cm^−1^) and amide II (1548 cm^−1^) bands, characteristic for proteins. However, for Coll and Coll+PLD+Glyc samples, the bands were slightly shifted to higher wavenumbers which correlated with the higher N1s atomic concentration percentage found in the XPS analysis compared to Coll+PLD values. The change of amide I band position to 1645 cm^−1^ could be ascribed to the presence of hydroxyl groups of adsorbed water within glycerol-modified grafts [23,24]. The shift of amide II band position to 1580 cm^−1^ could be speculated to be related to impurities in glycerol (containing COO^−^ groups) which were absent in pure glycerol [25,26] and which match with the XPS results. Thus, the collagen layer was demonstrated to remain on fibrous prostheses after the process of PLD coating which is important in view of the principal feature of the grafts—lack of permeability to blood. Presence of PLD was impossible to detect because primary amine groups which are characteristic for catecholamine compounds are likely to be overlapped by amide bands of collagen. The bands attributed to glycerol were observed at 921 cm^−1^ (O-H bending vibrations), 993 cm^−1^ (C-O stretching vibrations in CH_2_OH), 1034 cm^−1^ (C-O-C group), 1107 cm^−1^ (C-O stretching vibrations in CHOH) and in the region 3600–3000 cm^−1^ (O-H stretching vibrations) [23,25,27]. These bands, suggesting glycerol presence, were shown for Coll and Coll+PLD+Glyc samples. In the spectrum of the Coll+PLD sample, most of these bands disappeared, suggesting that at least a major amount of glycerol was eluted from the prostheses during PLD coating procedure (Figure 1c).

**Figure 2:**
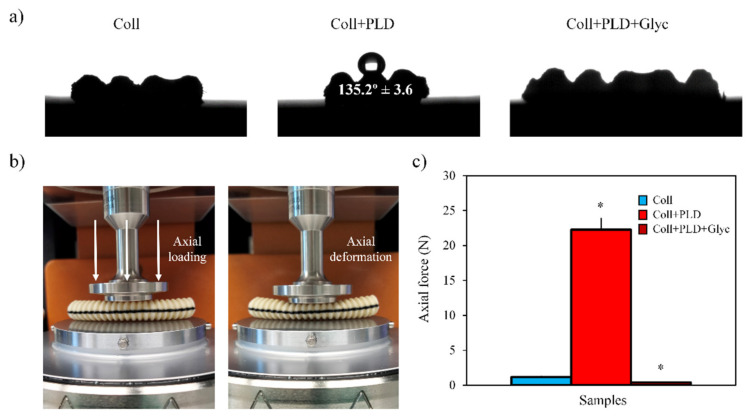
(**a**) Contact angle profile images of the Coll, Coll+PLD and Coll+PLD+Glyc vascular grafts. (**b**) Coll vascular graft placed between the parallel plates of the rheometer before and after applying axial loading force to deform the sample. (**c**) Axial force at 50% deformation for Coll, Coll+PLD and Coll+PLD+Glyc samples. (*) symbol indicates statistically significant differences for Coll+PLD and Coll+PLD+Glyc samples in comparison to the Coll sample.

From another point of view, the influence of the PLD coating and glycerol soaking process on the physical properties of the studied samples was evaluated focusing on their hydrophilicity and elastic behavior. Regarding the hydrophilicity of the vascular grafts, the contact angle of 10 µL water droplets was measured on the surface of Coll, Coll+PLD and Coll+PLD+Glyc samples (Figure 2a). Coll and Coll+PLD+Glyc vascular grafts displayed similar hydrophilic behavior since, after deposition, the water droplet was quickly absorbed within <1 s. This could be attributed to the presence of glycerol, which has low surface tension, favoring the wettability of the material. In contrast, in Coll+PLD samples, the water droplet was not absorbed and remained on the surface of the sample, showing a contact angle of 135.2° ± 3.6, typical of hydrophobic surfaces.

Regarding the elastic behavior, the force required to cause an axial deformation of 50% in all the studied vascular grafts was determined using a highly sensitive load cell in the rheometer (Figure 2b). In this experiment we confirmed the increase of the stiffness caused by the PLD coating, since the force required to deform the Coll+PLD samples (≈22 N) was around 19 times higher than Coll samples (≈1.17 N) (Figure 2c). In addition, during the compression, Coll+PLD samples denoted brittleness as they suffered visible cracks and, after applying the axial force, the samples were not able to recover the initial shape. In contrast, the Coll samples, in addition to lower stiffness, also displayed an elastic behavior since Coll samples were not cracked and were able to recover the initial state. Then, thanks to the reintroduction of glycerol into the vascular grafts structure in Coll+PLD+Glyc, the samples restored the stiffness and flexibility achieving similar axial force values to Coll (≈0.4 N for Coll+PLD+Glyc). Overall, analyzing the data obtained from the material characterization, we determined that the glycerol soaking process was able to restore the chemical and physical properties in Coll+PLD+Glyc vascular grafts, obtaining characteristics very close to the pristine Coll samples.

**Figure 3:**
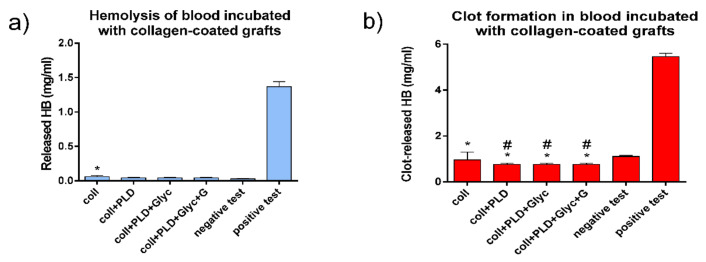
Hemolysis (**a**) and clot formation (**b**) in blood incubated with Coll, Coll+PLD and Coll+PLD+Glyc grafts. (*) symbol indicates statistically significant results between negative test and all samples; (#) symbol indicates statistically significant results between Coll and the rest of the samples, according to one-way ANOVA with post-hoc Tukey’s test (*p* < 0.05).

After performing the physicochemical characterization of all samples, we evaluated the biological safety of the studied vascular grafts. Biological safety of implantable materials concerns many aspects of biomaterial–human body interactions. In this sense, vascular prostheses are designed for continuous contact with circulating human blood. Therefore, blood reaction to the contact with modified and control prostheses was evaluated in terms of blood hemolysis and clot formation. All tested prosthesis samples, Coll, Coll+PLD and Coll+PLD+Glyc, were found relatively safe for evaluated blood parameters. With respect to hemolysis, all PLD-coated prostheses were comparable to negative test, whereas the Coll graft induced slight but significantly different blood hemolysis (Figure 3a). With respect to clot-forming dynamics, the lower the amount of hemoglobin released from erythrocytes not entrapped within a clot, the higher the blood clotting capacity. As shown in Figure 3b, all the grafts caused significantly different but slight increases of clot-forming rate in comparison with negative test. Moreover, all three PLD-coated samples slightly induced clot formation (this induction was also significantly different) when compared to Coll grafts (Figure 3b), which is promising in relation to reduction of wound bleeding directly after the implantation. Overall, neither PLD-coating nor glycerol addition was found to be harmful for interactions between the grafts and human blood.

**Figure 4:**
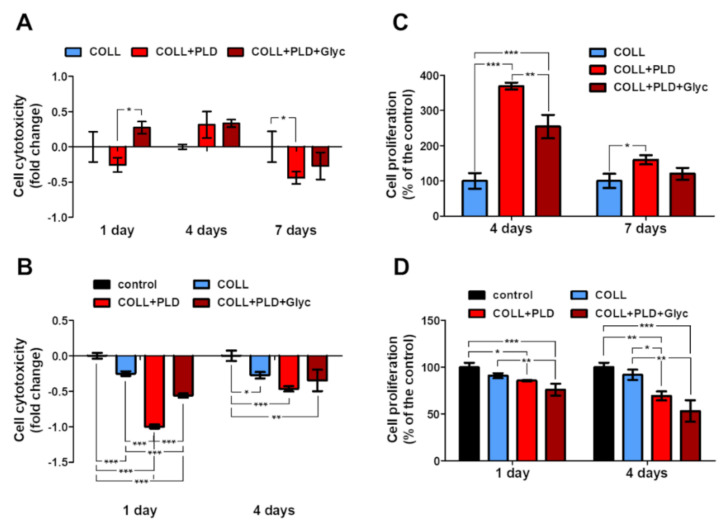
Effect of the prostheses on HUVEC cells: (**A**,**B**) prosthesis cytotoxicity evaluated with LDH assay towards cells grown on them for 1, 4 and 7 days (direct test; **A**) and cytotoxicity of the extracts (indirect test; **B**); (**C**,**D**) proliferation of the cells grown on the biomaterials (direct test; **C**) and incubated with the prosthesis extracts (indirect test, **D**); statistical significance was indicated with (*) symbol; *—*p* < 0.05, **—*p* < 0.01, ***—*p* < 0.001, one-way ANOVA, Tukey’s post hoc test.

Another important aspect of human–body–implant interactions concerns the cytotoxicity against a target cell line. For vascular prostheses, human endothelial cells (HUVECs) are the most appreciated model. Therefore, viability and proliferation of HUVECs was evaluated for Coll, Coll+PLD and Coll+PLD+Glyc graft samples. The HUVEC cells grew on all prostheses without harmful effects on them—no significant cytotoxicity was observed in almost all cases (excluding Coll+PLD+Glyc at 1 day incubation) (Figure 4A,B). On the 4th day a slight rise in cytotoxicity was noted, but no value was statistically significant (cytotoxicity higher by 8.6% and 2.7% than in the control, exerted by Coll+PLD and Coll+PLD+Glyc prostheses, respectively). By the 7th day, cytotoxicity of the Coll+PLD and Coll+PLD+Glyc prostheses was lower than Coll. Furthermore, a significant decrease in cytotoxicity was noted on the 7th day of growth on Coll+PLD prosthesis (cytotoxicity lower by 22.2% compared to the control (Coll) prosthesis, *p* = 0.0418) (Figure 4A). Such an event (higher cytotoxicity at 4th and lower at 7th day) may have occurred due to adaptation of the cells, previously grown as 2D culture, to the new conditions of 3D culture. Besides the growth of the cells on the prostheses (direct tests), the effect of the prosthesis extracts was evaluated (indirect tests). Cells were incubated with medium previously incubated with prostheses or without them (used in the indirect analyses as control). Significant decrease in LDH level was observed, along with slight decrease in proliferation. No prosthesis extract exerted a cytotoxic effect towards HUVEC cells. Furthermore, the cytotoxicity of Coll+PLD and Coll+PLD+Glyc extracts was lower than in the case of Coll extract. After 1-day incubation, Coll+PLD and Coll+PLD+Glyc graft extracts caused a significant decrease in cell cytotoxicity (by 99.9% and 56.0% of the control, respectively, with *p* < 0.0001), compared to both control and Coll prosthesis’ (cytotoxicity decrease of Coll by 25.1% compared to the control) extracts. After 4-day incubation, observed cytotoxicity decrease was lower (decreased by 46.6 and 34.7% compared to the control) than after 1-day; however, it was still lower than in control and Coll prosthesis (decrease by 27.4% in reference to the control) variants (with *p* = 0.0013) (Figure 4B).

The proliferation of the cells on the Coll+PLD prostheses was induced compared to the Coll. Initially, the cell divisions were boosted, which was noted as a statically significant rise in proliferation at the 4th day (to the 396.6 ± 9.4% and 254.5 ± 33.5% of the control, in case of Coll+PLD and Coll+PLD+Glyc prostheses, respectively; *p* < 0.0001). With time, the proliferation acceleration was slowed down, leading to the proliferation rate at 7th day of 160.1 ± 13.2% and 120.4 ± 16.8% compared to the control, of the cells grown on Coll+PLD and Coll+PLD+Glyc prostheses, respectively (with statistically significant results in the case of Coll+PLD prosthesis with *p* = 0.0130) (Figure 4C). However, the rate of proliferation of the HUVEC on Coll+PLD and Coll+PLD+Glyc prostheses was still higher than on Coll. The proliferation of the HUVEC cells incubated with prosthesis extracts was lower than cells incubated with control extract. A slight but statistically significant decrease in cell proliferation was noted: to the 85.7 ± 0.7% and 75.9 ± 6.4% of the control (*p* = 0.0007), exerted by Coll+PLD and Coll+PLD+Glyc extracts, respectively, after 1-day incubation, and to the 69.3 ± 4.8% and 53.2 ± 11.5% of the control (*p* = 0.0009), respectively, after 4-day incubation. The Coll prosthesis extract exerted a decrease in proliferation (to the 90.9 ± 2.7% and 91.9 ± 5.6% of the control, after 1 and 4 days, respectively); however, the values were not statistically significant (Figure 4D).

The modified prostheses exerted no cytotoxic effect and induced proliferation of the HUVEC cells. It has been demonstrated that modification of commercially available collagen-sealed prostheses with polymers or proteins may be beneficial for the cells, i.a. through improvement of the cell adhesion or proliferation [28,29,30]. The lowest LDH level and highest proliferation ratio were observed in the case of the Coll+PLD prosthesis. As mentioned before, glycerol was washed off from the biomaterial during the modification step. The compound is, however, present on Coll and Coll+PLD+Glyc prostheses. This may indicate the negative effect of the glycerol on HUVEC cells. The harmful effect of the glycerol on cells is carried mainly through alteration in osmotic forces and has been demonstrated before on various cellular models [31,32]. However, it seems that modification of the commercially available prosthesis with PLD reversed the harmful effect of the glycerin, resulting in lower cytotoxicity and higher proliferation (Coll+PLD+Glyc vs. Coll). Slightly different results were obtained in MTT indirect test. In general, the differences between direct and indirect tests are a consequence of differences between 3D and 2D cultures. Furthermore, culture for MTT indirect test was continued for 4 days without medium exchange, which may have resulted in depletion of the medium of nutrients and accumulation of products of metabolism.

**Figure 5:**
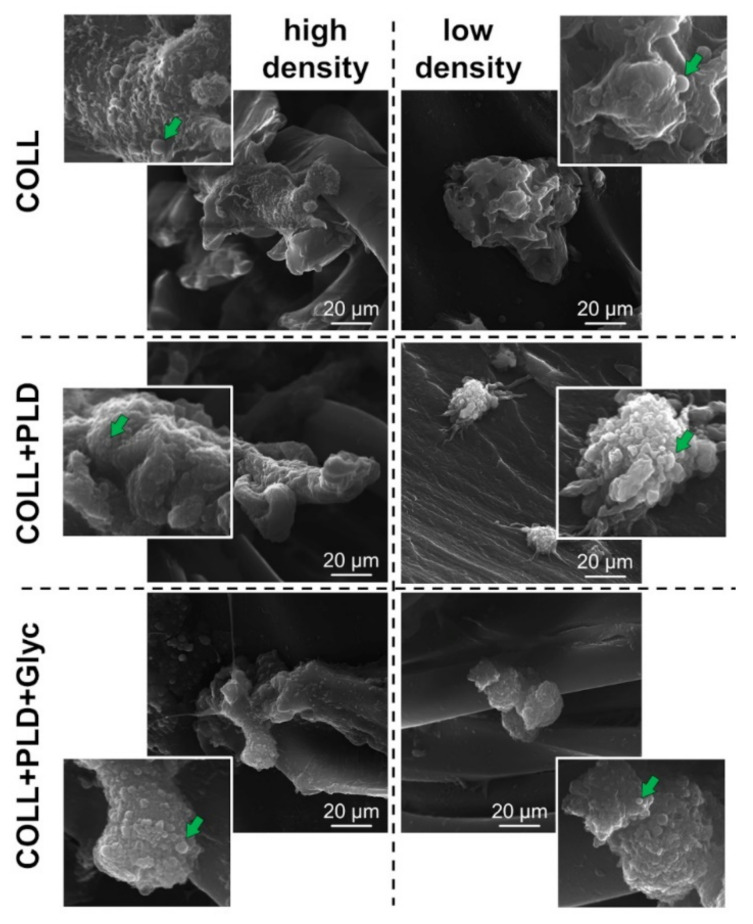
Morphology of the HUVEC cells at sites with high (**left column**) and low (**right column**) density, grown on the Coll (**upper row**), Coll+PLD (**middle row**) and Coll+PLD+Glyc (**bottom row**) prostheses for 4 days—SEM; measurement bars indicate 20 µm, magnification: 5000×, green arrows indicate potential extracellular vesicles.

Morphology of the HUVEC cells grown on the prostheses for 4 days was evaluated. Pictures of high and low density of the cells were taken using SEM technique (complete set of SEM images is available in Supporting Information file). No significant changes were observed in comparing cells grown on Coll+PLD and Coll+PLD+Glyc prostheses with ones cultured on Coll prostheses. However, cells present at sites with lower cellular density grown on Coll+PLD and Coll+PLD+Glyc prostheses appeared to possess rounder structures, presumably extracellular vesicles, compared to the cells grown on Coll prostheses (for the reference images, see: [33,34,35] (Figure 5, right column). It has been demonstrated that production of the EVs and microvesicles by endothelial cells and their progenitors may be beneficial for activation, growth and survival of the endothelial cells and vascularization process [36,37]. Coll+PLD and Coll+PLD+Glyc prostheses, which turned out to be more suitable for HUVEC cell growth compared to the control Coll prosthesis, induced production of the vesicles (Figure 5, right column). Nevertheless, no harmful effect was noted in the case of each prosthesis.

**Figure 6:**
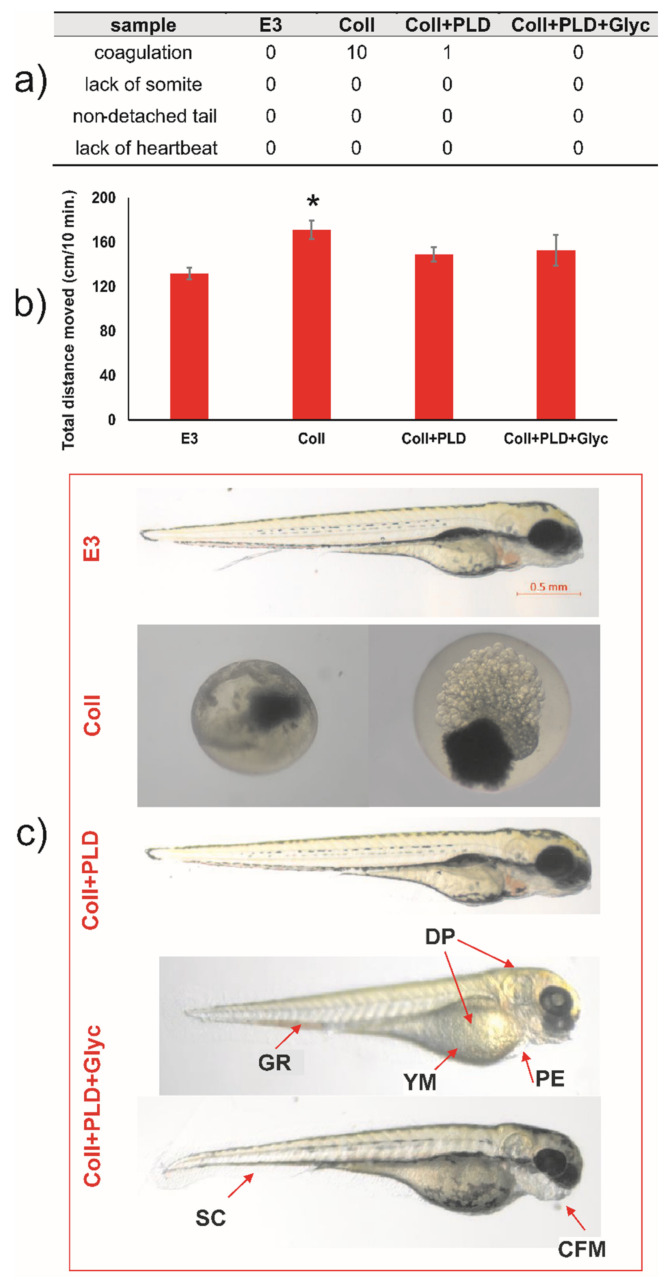
Evaluation of toxic and neurotoxic effect of extracts of Coll, Coll+PLD and Coll+PLD+Glyc grafts in Danio rerio model. (**a**) Apical observation of acute toxicity; (**b**) locomotor activity of 5 dpf (days post-fertilization) zebrafish larvae; (**c**) morphological changes at 96 hpf (hours post-fertilization) zebrafish larvae (SC—spinal curvature, CFM—craniofacial malformation; PE—pericardial oedema; YM—yolk malformation; DP—depigmentation; GR—growth retardation). (*) symbol indicates statistically significant results between control E3 medium and all samples, according to one-way ANOVA with post-hoc Tukey’s test (*p* < 0.05).

Results of experiments on the Danio rerio model gave interesting results concerning the toxicity of the modified vascular prostheses. During 96 h post-fertilization, the lack of coagulation, tail detachment, development of somites, and heartbeat was observed for negative E3 control. For extracts of prostheses samples, the coagulation was noticed: 10 per 10 embryos treated with extract of Coll, 1 per 10 embryos in extract of Coll+PLD and 0 per 10 in extract of Coll+PLD+Glyc (Figure 6a). Thus, extract of Coll (pristine graft) was shown to be highly embryotoxic while for that of Coll+PLD and Coll+PLD+Glyc it was shown to be highly safe.

For embryos incubated in extracts of Coll+PLD and Coll+PLD+Glyc grafts, the appearance of morphological and physiological abnormalities was controlled as a marker of cytotoxic effect. There was no incidence of morphological and physiological abnormalities of zebrafish during 4 days of incubation and also in extract of Coll+PLD, suggesting the safety of poly(L-DOPA) modification. Extract of Coll+PLD+Glyc grafts induced craniofacial malformation, (3/10), pericardial oedema (7/10), spinal curvature (1/10), yolk sac malformation (7/10), depigmentation (7/10) and growth retardation (4/10) (Figure 6a,c). In LLA, extract of pristine prostheses (Coll) influenced the locomotor activity of 5 dpf zebrafish in a statistically significant way one-way ANOVA: F (4,88) = 3.626; *p* = 0.0123). Thus, we cannot exclude the neurotoxic effects of extract of Coll graft (Figure 6b). The other studied extracts did not exert this effect. Overall, the results observed for Danio rerio model confirmed our observations for HUVECs cultures suggesting that PLD-coating generally reduced the toxicity of Coll grafts and that glycerol-regeneration of Coll+PLD grafts is less harmful for both models than pristine grafts (Coll).

On the basis of these experiments, it is quite clear that pristine collagen-sealed vascular grafts are not entirely safe in zebrafish model. Despite their approval for commercial use in medicine, the Coll graft showed strong pro-coagulation activity against fish embryos under stringent cytotoxicity assessment conditions of the performed tests (concentrated extract possessed by prosthesis incubation with E3 medium in proportion 0.1 g/1 mL). Comparison of the results obtained for Coll and Coll+PLD grafts suggested that glycerol may exert cytotoxic effect against Danio rerio embryos. As glycerol constituted approximately 50% weight of Coll and Coll+PLD+Glyc grafts, the extracts prepared for Danio rerio tests contained approx. 5% of glycerol. It was reported that 0.5% glycerol is the highest concentration safe for Danio rerio 1 hpf (hour post-fertilization) embryos [38]. Moreover, 5% glycerol reduced the survival of the embryos with the rate related to their age—from 0% of survival for 1 hpf embryos to 60% of survival for 36–48 hpf embryos [38]. Similarly, Maes et al. [39] showed the increasing tolerance of Danio rerio embryos to glycerol depending on their age: 4 hpf embryos tolerated 1.5% glycerol while 5–7 dpf tolerated above 2.5% glycerol. They also demonstrated that 1 hpf embryos treated with 2.5% glycerol displayed anterior-posterior axis truncation with no extended tail, *cardia bifida* and u-shaped somites [39]. However, both Coll and Coll+PLD+Glyc contained similar amounts of glycerol but the Coll graft was much more harmful for zebrafish embryos than Coll+PLD+Glyc ones (Figure 6). It is therefore possible that some post-production impurities in the pristine graft were responsible for its higher toxicity in comparison with the Coll+PLD+Glyc graft. Nevertheless, one should remember that pristine vascular prostheses were designed for the implantation into blood vessels in mature human organism and harmful agents are likely to be eluted/diluted by blood circulating around the implant, thus reducing their negative effect.

The second of the goals of this work was to verify whether PLD coating on collagen-sealed vascular grafts can serve as a platform for further immobilization of antibiotics to protect the grafts against bacterial adhesion and growth. Pilot experiment confirmed this hypothesis.

**Figure 7:**
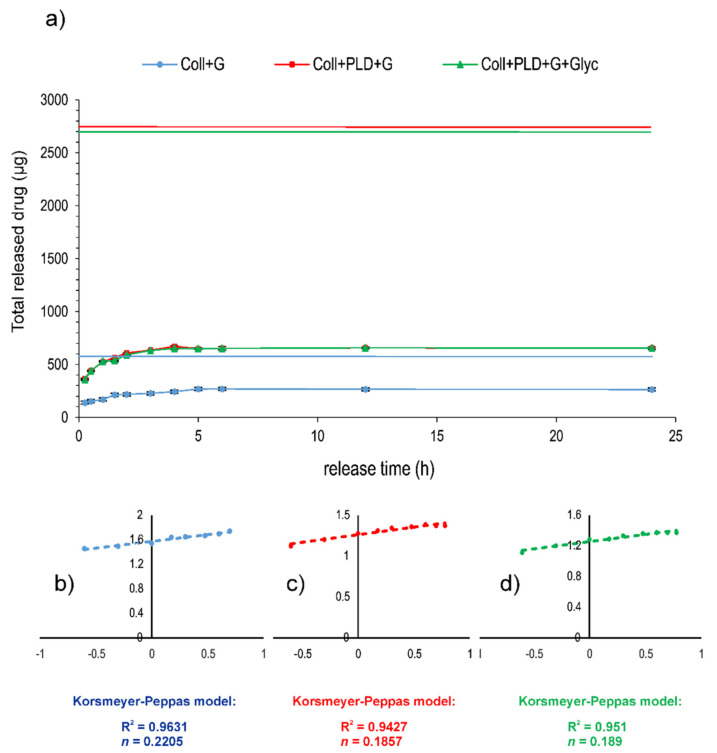
Profiles (**a**) and parameters (**b**–**d**) of gentamicin release in closed-loop system from Coll+G (**b**) Coll+PLD+G (**c**) and Coll+PLD+G+Glyc (**d**) grafts bound with the drug. Flat solid lines (**a**) indicate the total amount of drug loaded into the release cells—blue for Coll+G, red for Coll+PLD+G and green for Coll+PLD+G+Glyc, respectively.

To evaluate the effect of PLD graft coating on antibiotic binding more extensively, gentamicin release from prostheses before and after PLD coating (Coll and Coll+PLD, respectively) was coupled with the drug by simple soaking in antibiotic solutions and subjected to the release test in a close-loop system. Moreover, Coll+PLD+G was additionally soaked in glycerol solution (producing Coll+PLD+G+Glyc sample). Coll+G, Coll+PLD+G and Coll+PLD+G+Glyc samples were loaded into the release units in similar quantities (approx. 0.5 g of dry prosthesis weight). However, the amount of immobilized antibiotic significantly differed: 822 and 5067 µg/g of prosthesis for Coll+G and Coll+PLD+G or Coll+PLD+G+Glyc, respectively. Thus, the amounts of gentamicin loaded into the cells also differed: 485, 2758 and 2728 µg for Coll+G and Coll+PLD+G or Coll+PLD+G+Glyc, respectively (Table 3). The profiles of drug release are presented in Figure 7a. They all suggest a quick and pore-dependent [40] mechanism of drug release. Drug release exponents (*n*) calculated for all samples on the basis of the Korsmeyer–Peppas model were 0.2205, 0.1857 and 0.189 for Coll+G, Coll+PLD+G and Coll+PLD+G+Glyc, respectively (Figure 7b–d). According to Rither and Peppas [41], *n* values lower than 0.5 indicate Fickian diffusion dissolution mode. The drug was released from the prostheses during the first 5 h of the process: in 55.17% of initial content for Coll+G but only in 23.6–23.9% of initial content from Coll+PLD+G and Coll+PLD+G+Glyc grafts (Table 3). Profiles of gentamicin release from Coll+PLD+G and Coll+PLD+G+Glyc samples were similar. Therefore, it seems that glycerol soaking did not affect the profile of gentamicin release from PLD-coated samples. The amount of antibiotic remaining on prostheses for Coll+G was equal to 397 µg/g of prosthesis but for Coll+PLD+G and Coll+PLD+G+Glyc it was 3855–3871 µg/g of prosthesis, therefore 10-fold more (Table 3). The fact that PLD-coated grafts contain high amounts of non-eluted antibiotic attached to the fibers may suggest the higher probability of Coll+PLD+G (and Coll+PLD+G+Glyc) graft protection against bacterial adhesion.

To summarize, significantly more gentamicin was bound and more drug remained stably attached to the prosthesis after the release test in the case of PLD-coated grafts compared to pristine prostheses. A similar observation was made for PLD-coated curdlan hydrogels coupled with antibiotic—approximately 5 times more drug was bound to PLD-coated hydrogel than to a pristine one [20]. Glycerol soaking, used to restore initial elasticity of the grafts, did not play a significant role in this phenomenon.

**Figure 8:**
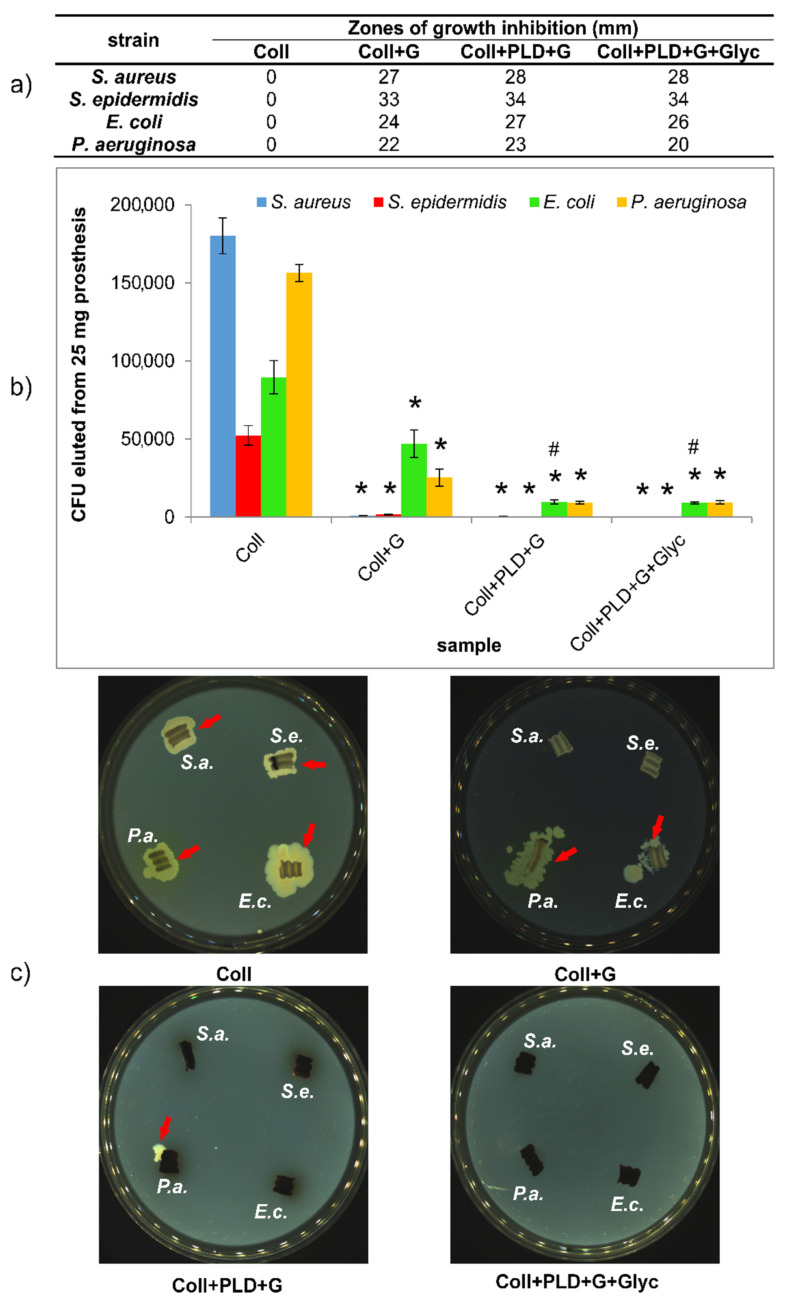
Short-term antibacterial activity of gentamicin-bound Coll+G, Coll+PLD+G and Coll+PLD+G+Glyc prostheses against four reference strains in comparison with Coll grafts. (**a**) Zones of bacterial growth inhibition; (**b**) bacterial adhesion expressed as the amount of prosthesis-adhered cells; (**c**) bacterial adhesion presented as spreading of adhered cells on solid culture medium. S.a.—*S. aureus*; S.e.—*S. epidermidis*; E.c.—*E. coli*; P.a.—*P. aeruginosa*. (*) symbol indicates statistically significant results for Coll prosthesis in comparison to the rest of samples, (#) symbol indicates statistically significant results for Coll+G prosthesis in comparison to Coll+PLD+G and Coll+PLD+G+Glyc samples, according to one-way ANOVA with post-hoc Tukey’s test (*p* < 0.05).

Careful evaluation of antibacterial properties of PLD-modified grafts is extremely important because collagen-sealed biomaterials are endangered per se (the collagen layer may serve both as an attachment surface and a nutrient for bacteria). Therefore, pristine Coll prosthesis was used as a reference in all experiments. Antibacterial activity of gentamicin-coupled pristine and PLD-coated prostheses was subjected to various tests. First, gentamicin diffusion test in agar medium was made, to evaluate how the prostheses were able to protect their close surroundings. The results showed, as expected, that Coll grafts did not show any growth inhibition zones for 4 Gram-positive and Gram-negative strains: *S. aureus, S. epidermidis, E. coli* and *P. aeruginosa*. Coll+G, Coll+PLD+G and Coll+PLD+G+Glyc samples caused the appearance of bacterial growth inhibition zones in the range 22–33 mm for non-modified graft and 20–34 mm for both PLD-modified samples (Figure 8a). Therefore, there is some positive effect of PLD-coating on bacterial safety of collagen-sealed prostheses. Glycerol did not cause any change in the size of growth inhibition zone around the samples for Gram-positive bacteria. However, the zones of *E. coli* and *P. aeruginosa* growth inhibition were slightly reduced for glycerol-soaked sample in comparison with Coll+PLD+G. This may result from the presence of glycerol as a nutrient for Gram-negative bacteria. The differences between PLD-treated and untreated grafts are small. However, it should be noted that only removable drugs act antibacterially in this growth inhibition test.

Another aspect of antibacterial properties of biomaterials is their ability to reduce the bacterial adhesion to their surfaces. This aspect was verified by incubation of the samples in bacterial suspension for 2 h and evaluation of their adhesion in two ways. First, graft-adhered bacteria were enzymatically detached using trypsin followed by their counting (by plating on agar medium method). Second, the grafts with adhered bacteria were placed directly on agar medium to verify whether the bacteria can divide, migrate into the surrounding medium and spread. The results showed that bacteria freely adhere to pristine Coll grafts (in amount 50,000–180,000 CFU/25 mg graft) and they can divide and spread when exposed to agar medium (Figure 8b,c). In the case of Coll+G graft, both Gram-positive strains adhered only to a small extent (933–1533 CFU), whereas *E. coli* and *P. aeruginosa* adhered to Coll+G graft in an amount reduced by approx. 50% and 80%, respectively, in comparison with Coll prostheses (Figure 8b). Interestingly, for both Gram-negative strains, PLD-coating caused additional reduction of graft-adhered bacterial cells in comparison with Coll+G (for *E. coli*, the reduction was significantly different) (Figure 8b).

A similar tendency was observed for bacterial migration from the grafts and spreading on agar medium. In the case of both PLD-modified grafts with gentamicin (without and with glycerin), no bacteria were attached to the samples with the exception of single *P. aeruginosa* colony. In contrast to Gram-negative strains, migration of Gram-positive bacteria to agar medium was not observed in the case of Coll+G samples (Figure 8c). In conclusion, all obtained results confirmed the beneficial effect of PLD-coating on antibacterial activity of collagen-sealed grafts. Thus, these results are in agreement with our previous data concerning PLD-treatment of polyester fibrous prostheses and their resistance to bacterial adhesion [19].

**Figure 9:**
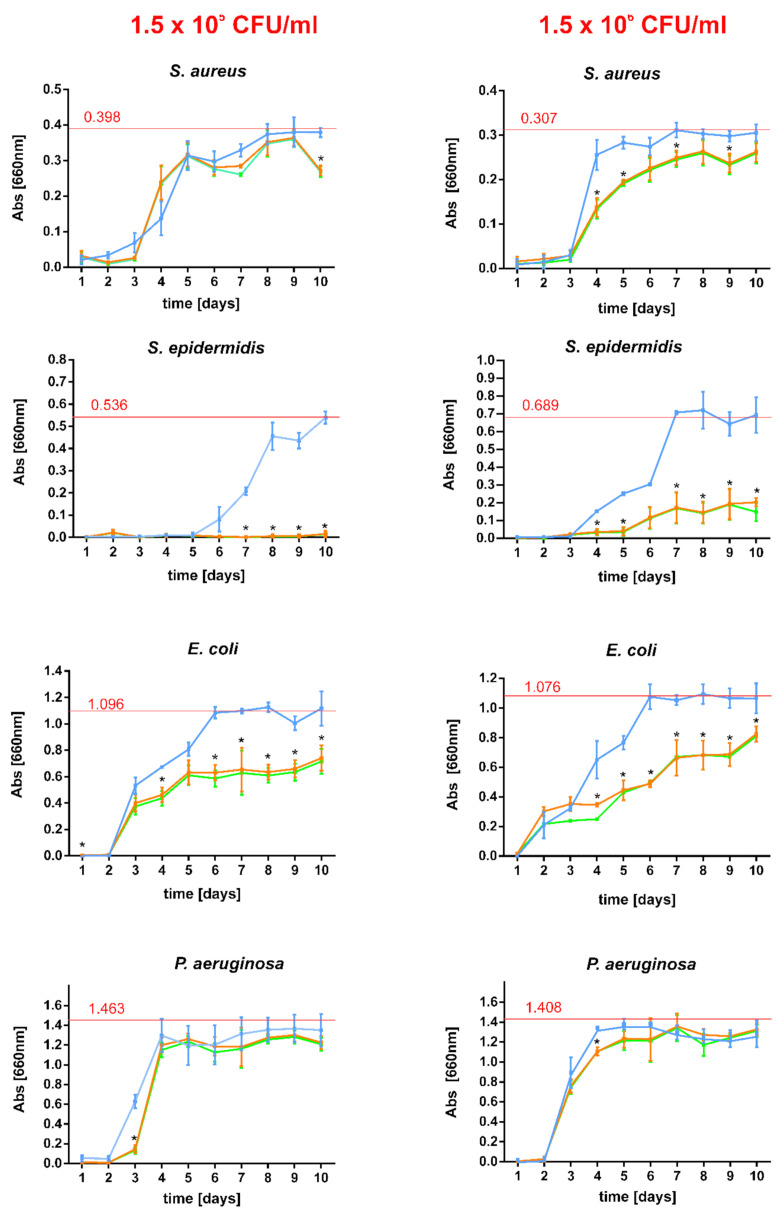
Long-term antibacterial activity of gentamicin-bound Coll+G, Coll+PLD+G and Coll+PLD+G+Glyc prostheses against four reference strains. The results are presented for extracts of prosthesis collected daily during a 10 day-long test and inoculated with bacteria (with initial density: 1.5 × 10^5^/mL or 1.5 × 10^6^/mL). The growth was expressed as culture absorbance at 660 nm and compared with averaged bacterial growth in control culture medium (100% growth—red line, with absorbance value marked over the line). Blue solid line—Coll+G; orange solid line—Coll+PLD+G; green solid line—Coll+PLD+G+Glyc. (*) symbol indicates statistically significant results for Coll+G prosthesis in comparison to Coll+PLD+G and Coll+PLD+G+Glyc, according to one-way ANOVA with post-hoc Tukey’s test (*p* < 0.05).

Although short-term resistance of biomaterials to bacterial attack is important, long-term response is also extremely essential. One must remember that implanted vascular grafts are permanently surrounded by circulating blood which cause constant elution of graft-bound antibacterial agent. Thus, gentamicin-bound pristine and PLD-coated grafts were subjected to drug elution for 10 days in subsequent buffer exchanges and antibacterial activity in collected extracts was evaluated against four reference strains (Figure 9). It was found that the antibacterial effect of collected extracts is: (i) strain-dependent and (ii) titer-dependent (in some cases). For *S. aureus*, PLD-modification of the prostheses only slightly reduced the rate of bacterial growth (the difference was significant for higher initial bacterial titer). However, this effect against *S. epidermidis* was much more distinct—in the lower initial titer of bacteria, their growth was completely inhibited while for the higher initial titer, their growth was significantly diminished (significantly different results). Similarly for the strain *E. coli*, PLD-coating caused partial reduction of bacterial growth in collected extracts (significantly different results). For *P. aeruginosa* this effect was much smaller and significantly indifferent (Figure 9). However, one must remember that both tested initial titers (1.5 × 10^5^ CFU/mL or 1.5 × 10^6^ CFU/mL) are very high and rarely appear in circulating blood. As reported elsewhere, in microbial infections, the typical target concentration in human blood is as low as 10^3^ CFU/mL [42]. Thus, the obtained results of bacterial growth reduction by the extracts collected during the experiment should be considered as highly promising in relation to the prevention of vascular graft infection [43].

## 3. Materials and Methods

### 3.1. Grafts Modification by PDA or PLD

FlowNit Bioseal^®^ collagen-sealed polyester knitted grafts were purchased from Jotec GmbH (Hechingen, Germany). The manufacturer declared that the collagen-sealing layer was prepared from bovine collagen type I and biologically harmless glycerol. L-DOPA (precursor of PLD) or dopamine (precursor of PDA) were supplied by Sigma-Aldrich (St. Louis, MO, USA), NaCl, CuSO_4,_ MgCl_2_ and MgSO_4_ by POCH, Gliwice (Poland). Pieces of prostheses (25 ± 2 or 50 ± 2 mg) were soaked in 10 mM Tris/HCl buffer pH 8.5, containing 2 mg/mL L-DOPA or dopamine, as described elsewhere [19]. In some cases, the mixture also contained 0.5 mM CuSO_4_ or/and 2.75% NaCl; 0.38% MgCl_2_ and 0.17% MgSO_4_. The process was performed for 24 h, at 25 °C (in pilot experiment) or at optimized temperature in further tests. For temperature optimization, the samples were coated at 25 °C, 37 °C and 50 °C. Modified samples were washed in deionized (DI) water to remove the unbound monomer and dried (37 °C).

For tests of cytotoxicity on endothelial cells, zebrafish toxicity and neurotoxicity and evaluation of antibacterial activity, the samples were sterilized in ethylene oxide (1 h at 55 °C, with subsequent 5 h aeration).

To restore the desired elasticity and hydrophilicity of modified grafts, pieces of the coated prosthesis were immersed for 10 min in 30% aqueous glycerol solution at 25 °C (5 mL per 0.2 g prosthesis) and then allowed to dry at 37 °C.

### 3.2. Antibiotic Immobilization

Immobilization of gentamicin (Sigma-Aldrich, USA) was performed by simple soaking of graft pieces (pristine and polycatecholamine-coated ones) in 1 mg/mL gentamicin solution in 0.1 M Britton-Robinson buffer pH 8.5 for 24 h, typically at 25 °C, 37 °C and 50 °C, depending on the particular experiment. Then the samples were washed twice in distilled water and dried at 37 °C. The amount of immobilized drug was calculated from gentamicin concentrations in the buffer before and after the process and evaluated quantitatively after drug derivatization with phthaldialdehyde (Sigma-Aldrich), as described elsewhere [21]. For comparison, the reference method of gentamicin immobilization on protein-sealed vascular prostheses using 0.5–2.5% glutaraldehyde (Chempur, Piekary Śląskie, Poland) was performed on FlowNit Bioseal^®^ collagen-sealed polyester knitted prosthesis, according to a procedure described elsewhere [5].

Samples of grafts obtained in optimized coating conditions and with immobilized gentamicin were selected for further experiments. They are listed in Table 1.

### 3.3. Characterization of Modified Prostheses

#### 3.3.1. FTIR Spectra

FTIR-ATR evaluation of graft chemical structure was performed using Vertex 70 spectrometer equipped with diamond crystal accessory and OPUS 7.0 software (Bruker, Billerica, MA, USA) at 4000–400 cm^−1^, spectral resolution 4 cm^−1^, 64 scans.

#### 3.3.2. X-ray Photoelectron Spectroscopy (XPS)

The chemical composition of the surface of the Coll, Coll+PLD and Coll+PLD+Glyc vascular grafts to assess the influence of the poly-(L-DOPA) coating and the glycerol soaking process was determined by X-ray Photoelectron Spectroscopy (XPS, SPECS Surface Nano Analysis GMbH, Berlin, Germany). Data were acquired in ultrahigh vacuum (5.0 × 10^−9^ mbar) using an XR50 Aluminum anode source operating at 300 W and a Phoibos 150 MCD-9 detector (D8 advance, SPECS Surface Nano Analysis GmbH, Berlin, Germany). The spectra were recorded at a pass energy of 20 eV with a step size of 1.0 eV for survey spectra and 0.1 eV for high resolution spectra. The recorded core levels C1s, O1s, N1s peaks were used as a reference. CasaXPS software (Casa Software Ltd., Teignmouth, UK) was used for the analysis to determinate the atomic elemental composition and the C1s peak deconvolution applying the manufacturer set of relative sensitivity factors. The relative error associated with the survey spectra XPS measurements is 0.5%.

#### 3.3.3. Surface Topography

The surface topography of the Coll, Coll+PLD and Coll+PLD+Glyc samples was investigated by Scanning Electron Microscopy (SEM) (Phenom XL Desktop SEM, PhenomWorld, Eindhoven, The Netherlands) operating at 5 kV. Due to the low conductivity of the samples, all vascular grafts were previously sputtercoated with carbon to avoid charging effects and to improve the secondary electron signal required for the topographic examination. Then, surface images of all samples were acquired at different levels of magnification.

#### 3.3.4. Contact Angle

The surface wettability of the Coll, Coll+PLD and Coll+PLD+Glyc vascular grafts was evaluated by measuring the contact angle of 10 µL water droplets deposited over the samples by using DSA100 Drop Shape Analyzer (Krüss, Hamburg, Germany).

#### 3.3.5. Mechanical Testing

The compression properties of the Coll, Coll+PLD and Coll+PLD+Glyc samples were determined by the axial force resistance parallel plate test in the rheometer (Hybrid Rheometer Discovery HR-2, TA Instruments, New Castle, DE, USA). To that end, the samples were placed onto the base of the rheometer and then the parallel plate was programmed to apply vertical displacement at 1 mm/min until reaching 50% deformation of the sample. Axial force was monitored during the whole compression process measured in Newtons (N).

### 3.4. Blood Compatibility Tests

Blood reaction to the contact with pristine and PLD-coated grafts was evaluated in the hemolysis and blood clot formation tests. Both these tests were performed on Coll, Coll+PLD and Coll+PLD+Glyc samples, weighing 50 mg ± 2 mg (in triplicate), as described elsewhere [19,20]. Briefly, citrated human blood was obtained from healthy volunteer (in accordance with KE-0254/258/2020 agreement of Bioethics Committee at the Medical University of Lublin, Poland). Total blood hemoglobin was 148 mg% and blood plasma hemoglobin concentration was 0.13 mg/mL, respectively (according to the reaction with Drabkin reagent and appropriate calibration curve, using BioTek Synergy H4 hybrid microplate reader; Thermo-Fisher Scientific, Waltham, MA, USA). As negative controls, 50 mg ± 2 mg of blood-neutral HDPE (high density polyethylene, Sigma-Aldrich, St. Louis, MI, USA) pieces were used. As positive controls, 0.1% Triton X-100 (Sigma-Aldrich, St. Louis, MO, USA) was used as hemolysis-inducing agent in the hemolysis test while non-activated Ca^2+^-free whole blood—in blood clot formation test.

Hemolysis was measured as a function of hemoglobin released from erythrocytes destroyed during 3 h contact between the prostheses samples and blood diluted 100-fold in PBS pH 7.4 (5 mL/50 mg of graft) at 37 °C. Clot formation was evaluated by incubation of the samples with 100 µL whole CaCl_2_-activated blood for 45 min at 37 °C, followed by measurement of hemoglobin in free erythrocytes not entrapped within the clot. In this test, there was an inverse relationship between the concentration of hemoglobin released from free erythrocytes not entrapped within a clot and the blood clotting capacity. In both tests, the amount of erythrocytes-released hemoglobin was evaluated using Drabkin reagent, as above. Statistically significant differences between negative control and various samples were considered at *p* < 0.0001, according to a one-way ANOVA with post-hoc Dunnett’s test (GraphPad Prism 8.0.0 Software, San Diego, CA, USA).

### 3.5. Endothelial Cell Cultures

#### 3.5.1. Cell Cultures

The analyses were carried on HUVEC cell culture (ATCC CRL-1730). The cells were cultured in F12K medium (ATCC), supplemented with 10% (*v*/*v*) FBS (Gibco), 0.1 mg/mL heparin (Sigma, St. Louis, MO, USA) and 30 mg/mL ECGS (endothelial cell growth supplement; Sigma, St. Louis, MO, USA), at 37 °C in humidified atmosphere with 5% CO_2_. For the analyses, cells at 3rd passage were trypsinized, counted and diluted to the desired density. The analyses were performed by direct (i.e., cells grown on the prostheses) and indirect (i.e., incubation of 2D cell cultures with prosthesis extracts) tests in triplicates, according to the ISO 10993-5 and 10993-12 standards.

#### 3.5.2. Growth of the Cells on the Prostheses

The graft samples were incubated for 24 h at 37 °C in complete medium (F12K with 10% (*v*/*v*) FBS, heparin and ECGS) in a 48-well plate. Afterwards, cell suspensions containing 5 × 10^4^ cells were added to each well. Cells were left for 24 h to adhere, and then incubated for 1, 4 or 7 days.

#### 3.5.3. Extract Preparation

The samples were cultured in a complete growth medium for 24 h at 37 °C with agitation. Volume of the medium was equal to the 1 mL per 0.1 g of the material (circa 250 µL per prosthesis, depending on its weight) and each extraction was carried in triplicates. Additionally, a medium was prepared, without being in contact with the prosthesis, in the same manner and used as control. After the incubation, the samples were collected and frozen at −80 °C.

#### 3.5.4. Cell Proliferation

Proliferation of the cells was evaluated with MTT method. In case of direct test, after 4 and 7 days of cell growth, 62.5 µL of 5 mg/mL MTT in PBS was added to each well. After 3 h, 250 µL of SDS was added to each well. The plates were read the next day using Microplate Reader (BioTek) at 570 nm. The results were presented as mean ±SD (*n* = 3) compared to the pristine (Coll) graft. For the indirect test, the HUVEC cells were seeded onto 96-well plates at 5 × 10^4^ cell/well. After 24 h, the medium was discarded and thawed extracts were poured into the wells, 50 µL per well, three repeats from each triplicate. As the control, medium without contact with prosthesis, but prepared in the same manner as extracts (incubated and frozen) was used. Afterwards, the cultures were incubated for either 1 or 4 days. Subsequently, MTT assay was performed, as described above (with the difference in volumes—12.5 µL of MTT and 50 µL of SDS were added to each well). The results were presented as mean ±SD (*n* = 3) compared to the control (medium incubated without prostheses).

#### 3.5.5. Prosthesis Cytotoxicity

To evaluate the cytotoxicity of the grafts (direct test), LDH assay was performed. The medium from the wells was collected on the 1st, 4th and 7th day and frozen at −80 °C. Afterwards, all samples were thawed and poured onto 96-well plates in triplicates and the assay was carried out according to the manufacturer’s manual (Sigma). The results were presented as mean ± SD (*n* = 3) compared to the pristine (Coll) graft. In case of indirect test, the cultures were prepared as in the MTT method. After the incubation, the LDH assay was performed. The results were presented as mean ± SD (*n* = 3) compared to the control (medium incubated without prostheses).

#### 3.5.6. Cell Morphology—SEM

Cells were grown on the prostheses for 4 days. Afterwards, the samples were fixed for 2 h with 4% glutaraldehyde in PBS and postfixed for 1 h in 1% osmium tetroxide in PBS, followed by dehydration in acetone series and air-drying in desiccator. Between each step, the samples were washed twice with PBS. After preparation, materials were mounted on SEM stubs and coated with conductive layer of gold in a vacuum sputter coated (Quorum Technologies Emitech K550X). The SEM procedure was performed with TESCAN VEGA 3 LMU microscope, working at 30 kV. The pictures representing cultures at sites of lower and higher cellular density were presented (magnification: 5000×).

### 3.6. Danio Rerio Embrio Toxicity (ET) and Locomotor Activity Assay (LAA)

Both tests were performed using Danio rerio of the AB strain (Experimental Medicine Centre, Medical University of Lublin, Poland), maintained in E3 embryo medium, at 28.5 °C, under standard aquaculture conditions, in an incubator (IN 110 Memmert, GmbH, Schwabach, Germany). The light/dark cycle was 14/10 h. Fertilized eggs were obtained by natural spawning. Both for ET and LLA experiments, the embryos were treated in extracts of Coll, Coll+PLD and Coll+PLD+Glyc grafts. The extracts were prepared by static incubation of the grafts with sterile E3 medium (37 °C, 24 h, proportion: 1 mL E3/0.1 g dry prosthesis weight). Immediately after the experiment, larvae were killed by immersion in 15 µM tricaine solution. The whole experiment was conducted in accordance with the National Institute of Health Guidelines for the Care and Use of Laboratory Animals and the European Community Council Directive for the Care and Use of Laboratory Animals of 22 September 2010 (2010/63/EU).

For the ET test, the collected embryos (each group of *n* = 15) in separate wells in 96-well plates were incubated with 200 µL of 2-fold diluted extracts or pure E3 medium. Apical observations of acute toxicity in zebrafish embryos 24–96 h post-fertilization (hpf) were performed on basis of coagulation, somite formation, tail detachment and heartbeat observation (using Zeiss Axio Vert stereomicroscope, ZEISS, Oberkochen, Germany). Image analysis was performed to determine the percentage of malformed embryos, taking into consideration the occurrence of morphological and physiological abnormalities. For LAA, a distance moved in a 10 min period (in cm) by 5 days post fertilization (dpf) larvae in a light condition was evaluated after 30 min incubation with tested extracts (each group of *n* = 20). Distance moved by larvae was evaluated using EthoVision XT 15 video tracking software (Noldus Information Technology b.v., Wageningen, The Netherlands). Statistical analysis was performed using Prism software (GraphPad Software, San Diego, CA, USA) and one-way analysis of variance (ANOVA).

### 3.7. Antibiotic Release

Gentamicin release from gentamicin-loaded grafts (Coll+G, Coll+PLD+G and Coll+PLD+G+Glyc) was performed in a closed-loop system using a USP 4 compliant flow-through cell tester Sotax CE1 (Sotax, Basel, Switzerland). Approximately 0.5 g of sample was loaded into the release cell for each graft. Test was performed in 35 mL PBS pH 7.4, 1 mL/min laminar flow rate, 37 °C. 0.3 mL portions of PBS were collected in specified time point and replaced by fresh PBS. In collected PBS samples, gentamicin was quantitatively evaluated, as described in Section 3.2, to determine the cumulative drug release profile. Drug release kinetics and mechanisms were determined using Korsmeyer–Peppas model, according to the equation:

M_t_/M_∞_ = kt^n^

where M_t_ is the amount of drug released from the composite in time t, M_∞_ is the accumulated released drug amount at time t→∞, and *k* and *n* are the kinetic constant and the release exponent, respectively. Nonlinear regression analysis was performed for drug release mechanism interpretation (using the Statistica 10 software; TIBCO software Inc, Palo Alto, CA, USA).

### 3.8. Antibacterial Tests

#### 3.8.1. Strains and Maintenance

Four bacterial strains (*Staphylococcus aureus* ATCC 25923, *Staphylococcus epidermidis* ATCC 12228, *Pseudomonas aeruginosa* ATCC 27853 and *Escherichia coli* ATCC 25922) were grown at 37 °C for 20–24 h in Mueller–Hinton Agar medium (Biomaxima, Poland). Then the bacteria were scratched and suspended in sterile physiological solution or Mueller–Hinton (M-H) broth (Biomaxima, Poland) to appropriate density.

#### 3.8.2. Pilot Test of Bacterial Growth Inhibition in Direct Contact

The test described in this section was performed on all graft samples synthesized during optimization of coating conditions. Sterile pieces of the grafts (50 ± 2 mg) were placed inside the wells of 12-well plate (Corning, New York, NY, USA) with 1 mL of MH broth and inoculated with *S. epidermidis* suspension to final density: 3 × 10^5^ CFU (colony forming units)/1 mL. The plates were incubated for 20–24 h (37 °C, 50 rpm, Innova 42, New Brunswick Scientific, Edison, NJ, USA). Every 24 h, graft samples were drained on sterile Whatman filter paper to remove the excess of medium and transferred to fresh wells with 1 mL of MH broth, inoculated as before. The experiment was performed until the appearance of visible growth of bacteria in the medium.

#### 3.8.3. Agar Plate Test

Test described in this section was performed on Coll, Coll+G, Coll+PLD+G and Coll+PLD+G+Glyc graft samples. Ø 7.5 mm wells were drilled in Mueller–Hinton Agar (Biomaxima, Lubelskie, Poland) on sterile Petri dishes. Then, 25 ± 2 mg pieces of the grafts were placed inside the wells. Then, the wells were filled with liquid Mueller–Hinton Agar medium which was allowed to set. Finally, 50 µL of bacterial suspension of each strain (3 × 10^7^ CFU/mL) was placed onto the agar and evenly spread. The plates were incubated at 37 °C for 20–24 h and bacterial growth inhibition zones were measured.

#### 3.8.4. Bacterial Adhesion Test

The test described in this section was performed on Coll, Coll+G, Coll+PLD+G and Coll+PLD+G+Glyc graft samples. Due to the strong auto-fluorescence of polyester fibers, the test was performed using trypsin to detach the adhered bacteria from grafts surfaces, as described earlier [18]. Briefly, four prostheses pieces (25 ± 2 mg) were incubated in the wells of 12-well plate with 2 mL of the suspension (3 × 10^8^ CFU/mL) of four bacterial strains. Plates were incubated at 37 °C for 2 h with agitation (100 rpm, Innova 42, New Brunswick Scientific, USA). Afterwards, the pieces of prostheses were transferred into 50 mL sterile Falcon tubes and carefully washed twice with 20 mL od 0.9% NaCl, to remove all free bacterial cells. Then one of four prosthesis pieces was placed onto M-H agar and incubated at 37 °C for 20–24 h, to check whether the adhered bacteria can divide, migrate into the surrounding medium and spread. The remaining three pieces were transferred into other 50 mL Falcon tubes and treated with 0.5 mL of 0.25% trypsin-EDTA solution (Sigma-Aldrich, St. Louis, MO, USA) for 15 min at 37 °C, to detach the adhered bacteria. After intense shaking on Vortex, 2 mL M-H broth was added to all tubes to “inactivate” trypsin. Finally, the resulting liquid was appropriately diluted and 50 µL of each dilution was plated (in triplicate) on M-H agar plates (using EasySpiral Dilute plater; Interscience, Saint Nom la Brétèche, France). The plates were incubated at 37 °C for 20–24 h and CFU were counted on each plate using Scan 300 counter (Interscience, France).

#### 3.8.5. Bacterial Growth Inhibition in Samples Extracts

The test described in this section was performed on Coll+G, Coll+PLD+G and Coll+PLD+G+Glyc graft samples. The samples were incubated in sterile M-H broth (in the proportion: 1 mL medium/0.1 g dry sample weight sample) at 37 °C, with agitation (50 rpm, Innova 42, New Brunswick Scientific, USA). The resulting extracts were collected daily and replaced by the same volume of fresh M-H broth for 10 days. The extracts were then dispensed into wells of 96-well plate (200 µL, in triplicate) and each well was inoculated with 10 µL of suspension of four bacterial strains (final density: 1.5 × 10^5^ CFU/mL or 1.5 × 10^6^ CFU/mL). Positive control of bacterial growth was tested separately for each strain in 200 µL of M-H broth, inoculated and treated as above. The plates were incubated for 20–24 h (37 °C, 100 rpm; on plate shaker DTS-4; ELMI, Newbury Park, CA, USA). After 24 h, absorbance of the media in the wells was measured at 660 nm using BioTek Synergy H4 hybrid microplate reader (Thermo-Fisher Scientific, Waltham, MA, USA). Inhibition of bacterial growth by prostheses samples was presented in comparison with the growth in positive controls.

## 4. Conclusions

The results obtained in this study lead to the conclusion that poly(L-DOPA)-coating (PLD-coating) on collagen-sealed knitted PET vascular grafts is a promising method of their functionalization. The modification takes place in mild conditions which leaves the collagen-sealing layer undamaged. However, it requires an additional step (quick soaking in glycerol solution) to restore desired elasticity and wettability of knitted prostheses. PLD-coating process reduced the toxicity of collagen-sealed commercial prostheses in vitro and in vivo (response of HUVECs and Danio rerio embryos), confirming their increased biological safety. Furthermore, the applied modification not only increased antibiotic-binding efficacy of the modified grafts (reducing bacterial growth and adhesion) but also reduced pro-hemolytic effects. On the basis of this study, we suggest that PLD-coating should attract more attention for modification of collagen-coated biomaterials. In particular, this modification may be recommended for improvement of polymeric biomedical devices of complex structures, such as textiles.

## Figures and Tables

**Table 1:** Sample codes.

Sample Code	Sample Description
Coll	Pristine graft
Coll+G	Pristine graft with gentamicin
Coll+PLD	Poly(L-DOPA)-coated graft
Coll+PLD+G	Poly(L-DOPA)-coated graft with gentamicin
Coll+PLD+Glyc	Poly(L-DOPA)-coated glycerol-regenerated graft
Coll+PLD+Glyc+G	Poly(L-DOPA)-coated glycerol-regenerated graft with gentamicin

**Table 2:** Surface elemental composition and atomic ratios of Coll, Coll+PLD and Coll+PLD+Glyc vascular grafts obtained by XPS, and C1s deconvolution.

	Atomic Concentration (%)			Atomic ratio		Atomic concentration from C1s deconvolution (%)		
	C1s	N1s	O1s	O/C	N/C	C-(C-H)	C-O, C-N	COO
Coll	78.68	5.27	16.04	0.2039	0.0670	71.43	18.54	10.04
Coll+PLD	85.77	3.39	10.3	0.1201	0.0458	73.48	18.67	7.86
Coll+PLD+Glyc	77.82	5.46	16.73	0.2149	0.0701	71.55	18.19	10.26

**Table 3:** Parameters of gentamicin release from tested samples.

	Sample		
	Coll+G	Coll+PLD+G	Coll+PLD+G+Glyc
Total amount of immobilized gentamicin (in µg/g prosthesis)	822	5067	5067
Total amount of cell-loaded prostheses (in g)	0.548	0.538	0.540
Total amount of cell-loaded gentamicin (in µg)	485	2758	2728
% of gentamicin released	55.17	23.6	23.9
Time of gentamicin release (h)	5	5	5
gentamicin released from 1 g prosthesis (in µg)	489	1195	1211
gentamicin remaining in 1 g prosthesis (in µg)	397	3871	3855

## Data Availability

Not applicable.

## Supplementary Materials

The following supporting information can be downloaded at: https://www.mdpi.com/article/10.3390/ijms23169369/s1. References [44,45,46] are cited in the Supplementary Materials.
