# Supplementary material for: Collagen-Sealed Polyester Vascular Prostheses Functionalized by Polycatecholamine Coatings
Source: Int J Mol Sci. 2022 Aug 19;23(16):9369. doi: 10.3390/ijms23169369 (PMC9409057; doi:10.3390/ijms23169369)
Supplement: Supplementary file 1 [file ijms-23-09369-s001.zip › ijms-1840860-supplementary.pdf]

Collagen-sealed polyester vascular prostheses functionalized by polycatecholamine coatings

Anna Michalicha[1], Cristina Canal[2,3], Albert Espona-Noguera[2,3], Mateusz Piet[4], Barbara Budzyńska[5], Anna Belcarz[1]

[1]Chair and Department of Biochemistry and Biotechnology, Medical University of Lublin, Chodzki 1, 20-093 Lublin, Poland
[2]Biomaterials, Biomechanics and Tissue Engineering Group, Materials Science and Engineering Department, and Research Center for Biomedical Engineering, Technical University of Catalonia (UPC), Escola d'Enginyeria Barcelona Est (EEBE), C/Eduard Maristany 14, 08019 Barcelona, Spain;
[3]Barcelona Research Center in Multiscale Science and Engineering, UPC, 08019 Barcelona, Spain;
[4]Department of Virology and Immunology, Maria Curie-Sklodowska University, Akademicka Str. 19, 20-033 Lublin, Poland
[5]Independent Laboratory of Behavioral Studies, Medical University of Lublin, Chodzki 4a, 20-093 Lublin, Poland

Results and discussion

In a pilot experiment we proved that coating of collagen-sealed vascular grafts using polycatecholamines is possible, both for dopamine and L-DOPA as monomers, as indicated by the appearance of a brownish black color in the coated grafts (Figure S1a). Visually, addition of Cu^{2+} salts resulted in darker color of the grafts which may suggest higher amount of polycatecholamine deposited on the prosthesis surface. The yield of gentamicin immobilization on coated grafts and their antibacterial efficacy was selected as a main criterion of the efficacy of the performed modification. Therefore, gentamicin was immobilized to such modified grafts via covalent bonds between free amino groups of the drug and catechol moieties of polymerized catecholamine [1,2]. For all tested conditions of modification, the amount of graft-immobilized gentamicin was at least 2-fold higher (1607-5108.5 µg/g of prosthesis) than for pristine grafts (820.5 µg/g of prosthesis). Comparing PDA and PLD coatings, drug immobilization was higher when PLD was used; it also increased when copper ions (0.5 mM) were added to the coating solution as a catalyst. In most cases, supplementation of the buffer with specified concentrations of Na^{+} and Mg^{2+} ions resulted in the increase of drug binding efficiency (Figure S1a). These results are in agreement with our earlier observations in experiments performed on polyester knitted prostheses modified with PLD [3]. Concentrations of NaCl, $MgCl_2$ and $MgSO_4$ used in experiments were similar as in seawater. It is therefore possible that salinity of environment during the PLD formation on the grafts is beneficial for this process. This hypothesis is supported by the earlier observation that presence of NaCl increased several times the thickness of PDA layer on some surfaces in comparison with the process without NaCl [4].

Also, antibacterial activity of gentamicin-bound grafts tested against *S. epidermidis* strain was higher when grafts were coated with polycatecholamine layer. Bacterial growth in medium incubated with these samples appeared on 4th-10th day of daily exchange which was an equivalent of 3-9 days of antibacterial protection. While for pristine graft, the bacterial growth was noted on 2nd day of the test (meaning that pristine graft soaked in drug solution was able to

protect its environment only for 1 day) (Figure S1b). Overall, collagen-sealed grafts coated with PLD in presence of NaCl, $CuSO_4$ and $MgCl_2$ exhibited the most promising results: 5108.5 µg gentamicin/1 g prosthesis and 9 days of bacterial growth inhibition (Figure S1a,b). This conclusion was similar to that withdrawn from the experiments performed on bare polyester knitted prostheses [3]. Therefore, this set of coating conditions was selected for further experiments.

Temperature generally affects the yield of immobilization process. For example, glucoamylase immobilization yield in cross-linked gelatin increases from 19.8 % at 25°C to 82.7 % at 60 °C [5]. Therefore, in our optimization experiments the effect of temperature (within the range 25-50 °C) on PLD-deposition on collagen-sealed prostheses and subsequent gentamicin binding was checked. It was observed that rise of temperature from 25 °C to 37 °C and 50 °C caused statistically different increase of amount of bound gentamicin (Figure S1c). Also, drug immobilization at 50 °C was more effective than at 37 °C. However, simultaneous destabilization of collagen+PLD layer was noted for the grafts treated at 50 °C (Figure 1c) – white areas suggesting the exposition of bare PET fibers were visible on the graft surface. It is known that collagen triple helices lose their stability at elevated temperatures. For collagen solution, the shift of triple helices to duplexes was observed at 35 °C [6]. Although immobilized compounds are usually more temperature-stable than the soluble ones, it is likely that treatment of collagen-sealed vascular grafts at 50 °C caused the denaturation and destabilization of collagen coating and its peeling off PET fibrous matrix. This phenomenon did not appear in case of the grafts modified at 37 °C. Therefore, despite the highest gentamicin immobilization yield observed for 50 °C, lower temperature of the prosthesis coating and subsequent drug immobilization (37 °C) was selected for further experiments.

Moreover, we decided to compare the efficacy of tested modification method with other procedure claimed effective for drug binding to protein-sealed vascular grafts. Ginalska et al. [7] reported the high efficacy of glutaraldehyde activation-based immobilization of gentamicin to gelatin-sealed knitted vascular grafts. Glutaraldehyde is a homobifunctional linker with two aldehyde groups. Its mechanism of action depends on formation of covalent bonds between its aldehyde groups and free amine groups of other molecules. In the case of our experiment, it could serve as a linker between collagen and gentamicin (as both these compounds contain free amino groups). Therefore, we wanted to verify whether this method of drug binding can be as effective as PLD-based method for collagen-sealed knitted vascular grafts. It was found that slight but statistically insignificant increase of amount of immobilized gentamicin was observed only for 0.5 % glutaraldehyde – 930 µg gentamicin/1 g prosthesis. Higher glutaraldehyde concentration (1.0-2.5 %) caused a significant (and significantly different) decrease (below 450 µg gentamicin/1 g of prosthesis) of immobilized drug amount (Figure S1d). This phenomenon may result from the preferential formation of internal crosslinking within collagen layer instead of reaction between collagen and gentamicin, which is possible in higher linker concentration. Nevertheless, this alternative activation method is not efficient for collagen-sealed grafts in comparison with PLD-based coating method.

References

[1] H. Lee, S.M. Dellatore, W.M. Miller, P.B. Messersmith, Mussel-inspired surface chemistry for multifunctional coatings, Science. 318 (2007) 426–430. https://doi.org/10.1126/science.1147241.Mussel-Inspired. 【14】

[2] A. Michalicha, K. Palka, A. Roguska, M. Pisarek, A. Belcarz, Polydopamine-coated curdlan hydrogel as a potential carrier of free amino group-containing molecules,

Carbohydrate Polymers. 256 (2021) 117524. 【21】
https://doi.org/10.1016/j.carbpol.2020.117524.

[3] A. Michalicha, A. Espona-Noguera, C. Canal, B. Budzyńska, M. Pięt, S. Przywara, J. Pawelec, A. Belcarz, Polycatecholamine and gentamicin as modifiers for antibacterial and blood-biocompatible polyester vascular prostheses, Materials Science and Engineering C. (2022) 112645. https://doi.org/10.1016/j.msec.2022.112645. 【19】

[4] J. Kuang, J.L. Guo, P.B. Messersmith, High ionic strength formation of DOPA-melanin coating for loading and release of cationic antimicrobial compounds, Advanced Materials Interfaces. 1 (2014) 1400145. https://doi.org/10.1002/admi.201400145.

[5] Z. Gan, T. Zhang, Y. Liu, D. Wu, Temperature-Triggered Enzyme Immobilization and Release Based on Cross-Linked Gelatin Nanoparticles, PLoS ONE. 7 (2012) 1–10. https://doi.org/10.1371/journal.pone.0047154.

[6] A. Marchand, M.F. Czar, E.N. Eggel, J. Kaeslin, R. Zenobi, Studying biomolecular folding and binding using temperature-jump mass spectrometry, Nature Communications. 11 (2020) 1–12. https://doi.org/10.1038/s41467-019-14179-x.

[7] G. Ginalska, M. Osinska, A. Uryniak, T. Urbanik-Sypniewska, A. Belcarz, W. Rzeski, A. Wolski, Antibacterial activity of gentamicin-bonded gelatin-sealed polyethylene terephthalate vascular prostheses, European Journal of Vascular and Endovascular Surgery. 29 (2005) 419–424. https://doi.org/10.1016/j.ejvs.2004.12.030. [5]

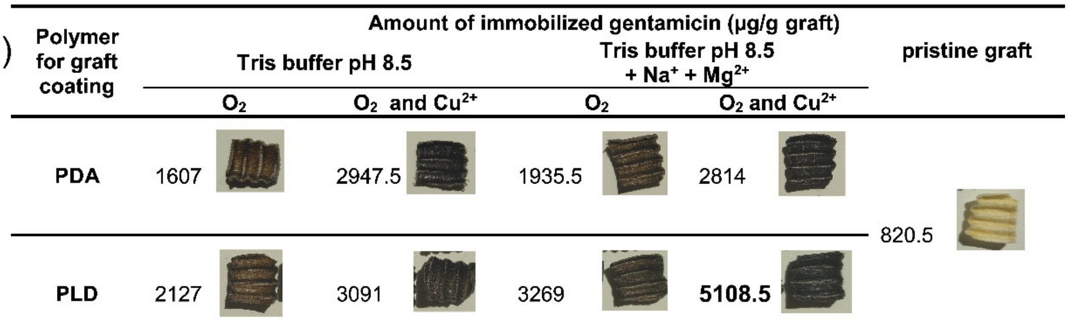

a)

Polymer for graft coating	Amount of immobilized gentamicin (µg/g graft)				
	Tris buffer pH 8.5		Tris buffer pH 8.5 + Na^+ + Mg^{2+}		pristine graft
	O_2	O_2 and Cu^{2+}	O_2	O_2 and Cu^{2+}	
PDA	1607	2947.5	1935.5	2814	820.5
PLD	2127	3091	3269	**5108.5**	

b)

Polymer for graft coating	Appearance of bacterial growth in extracts of the gentamicin-coupled grafts (in days)				
	Tris buffer pH 8.5		Tris buffer pH 8.5 + Na^+ + Mg^{2+}		pristine graft
	O_2	O_2 and Cu^{2+}	O_2	O_2 and Cu^{2+}	
PDA	6	6	6	7	2
PLD	4	4	7	**10**	

c)

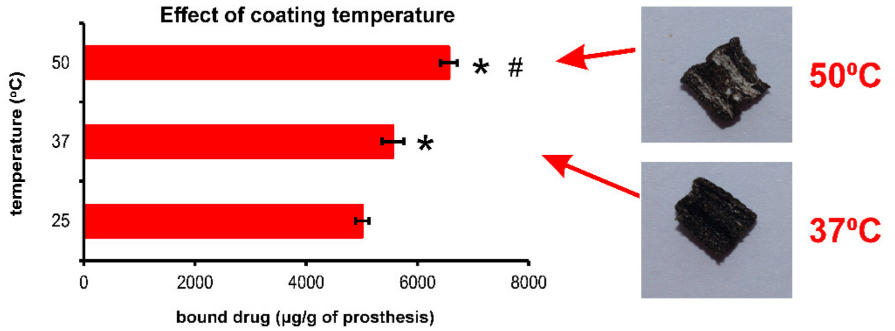

d)

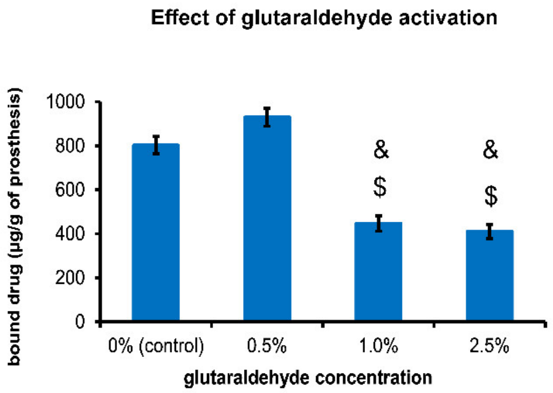

Figure S1. Optimization of polycatecholamine (PDA – polydopamine, PLD – poly(L-DOPA)) coating conditions on antimicrobial activity of collagen-sealed vascular prostheses. a) Amount of the immobilized drug and macrophotography of Coll (pristine graft) and Coll+PDA or Coll+PLD (polydopamine or poly(L-DOPA)-coated graft), using different catalysts and additives; b) *S. epidermidis* growth in direct contact with the prostheses coated in different conditions; c). Effect of coating temperature (for the process using PLD, Cu^{2+}, Na^+ and Mg^{2+}) on gentamicin binding to the prostheses and macrophotography of the grafts coated at 37 ºC and 50 ºC; d) Effect of glutaraldehyde (0.5-2.5 %) activation on gentamicin binding to Coll grafts. (*) symbol indicates statistically significant results for 37 ºC and 50 ºC in comparison to 25 ºC, (#) symbol indicates statistically significant results between 37 ºC and 50 ºC, ($) symbol indicates statistically significant results between 0.5 %, 1 % and 2.5 % glutaraldehyde in comparison with 0 % glutaraldehyde; (&) symbol indicates statistically significant results between 0.5 % in comparison with 1 % and 2.5 % glutaraldehyde, according to one-way ANOVA with post-hoc Tukey's test ($p < 0.05$).

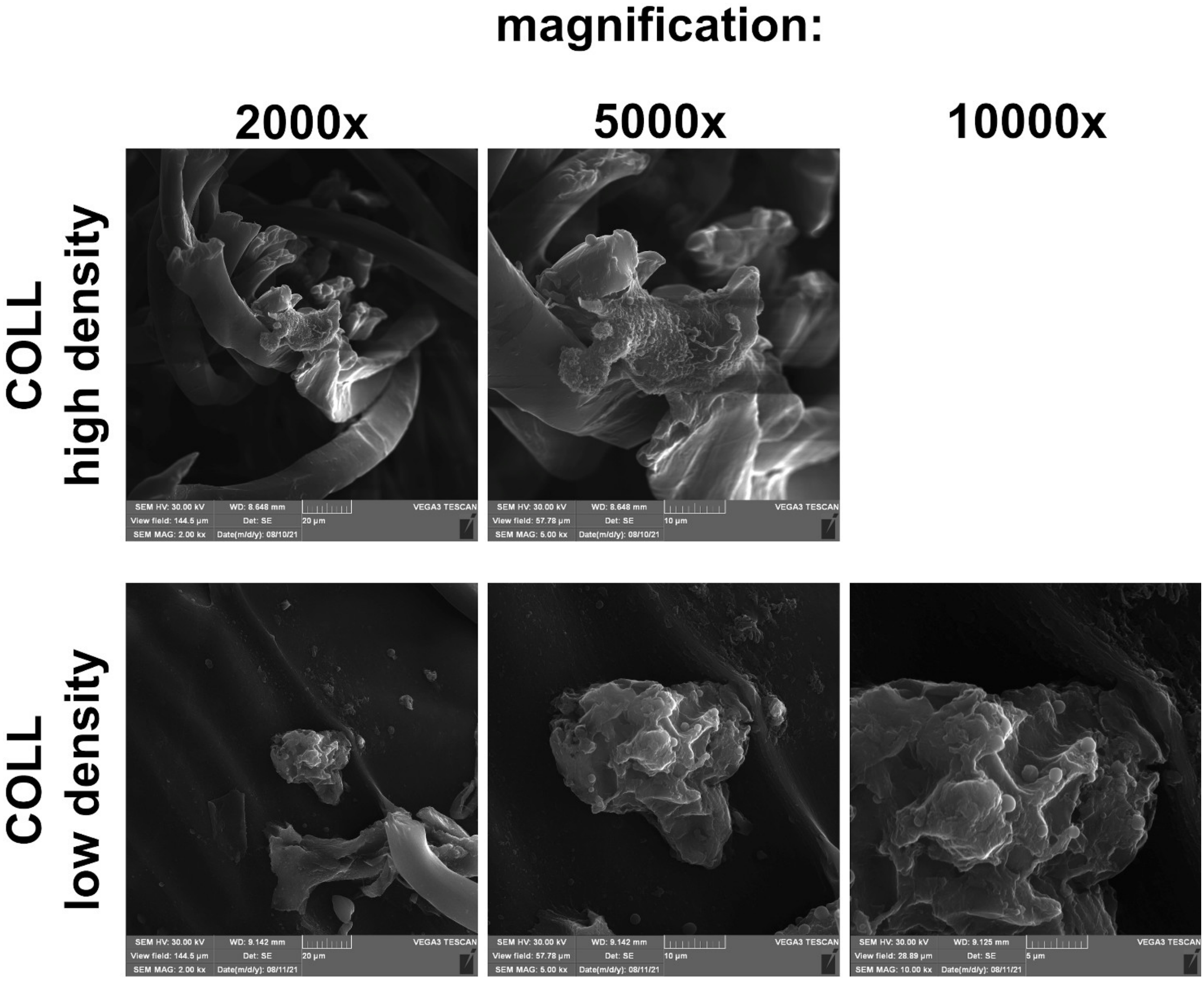

Figure S2. Uncropped and not rotated images of the HUVEC cells at sites with high and low density, grown on the Coll prostheses for 4 days – SEM

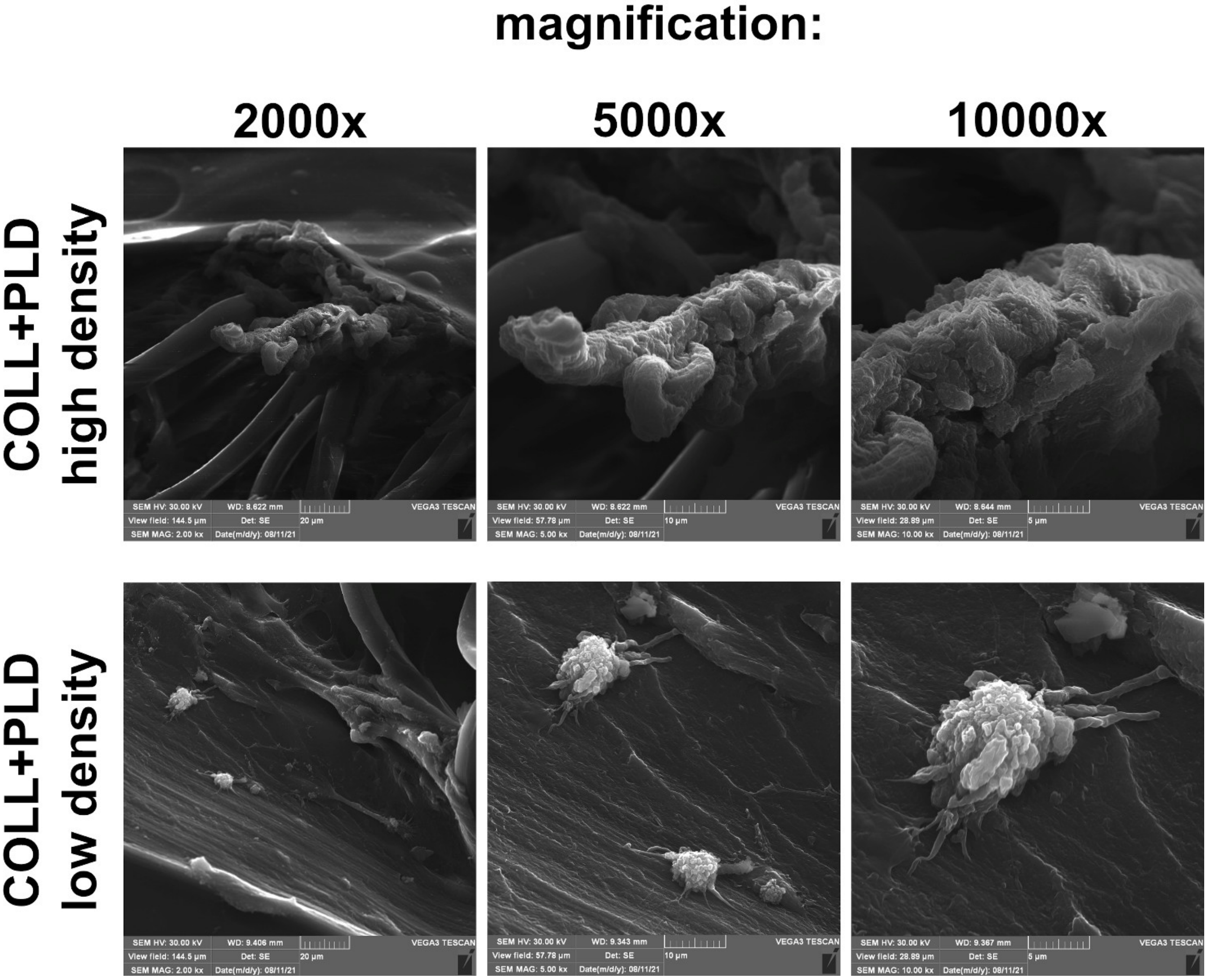

Figure S3. Uncropped and not rotated images of the HUVEC cells at sites with high and low density, grown on the Coll+PLD prostheses for 4 days – SEM

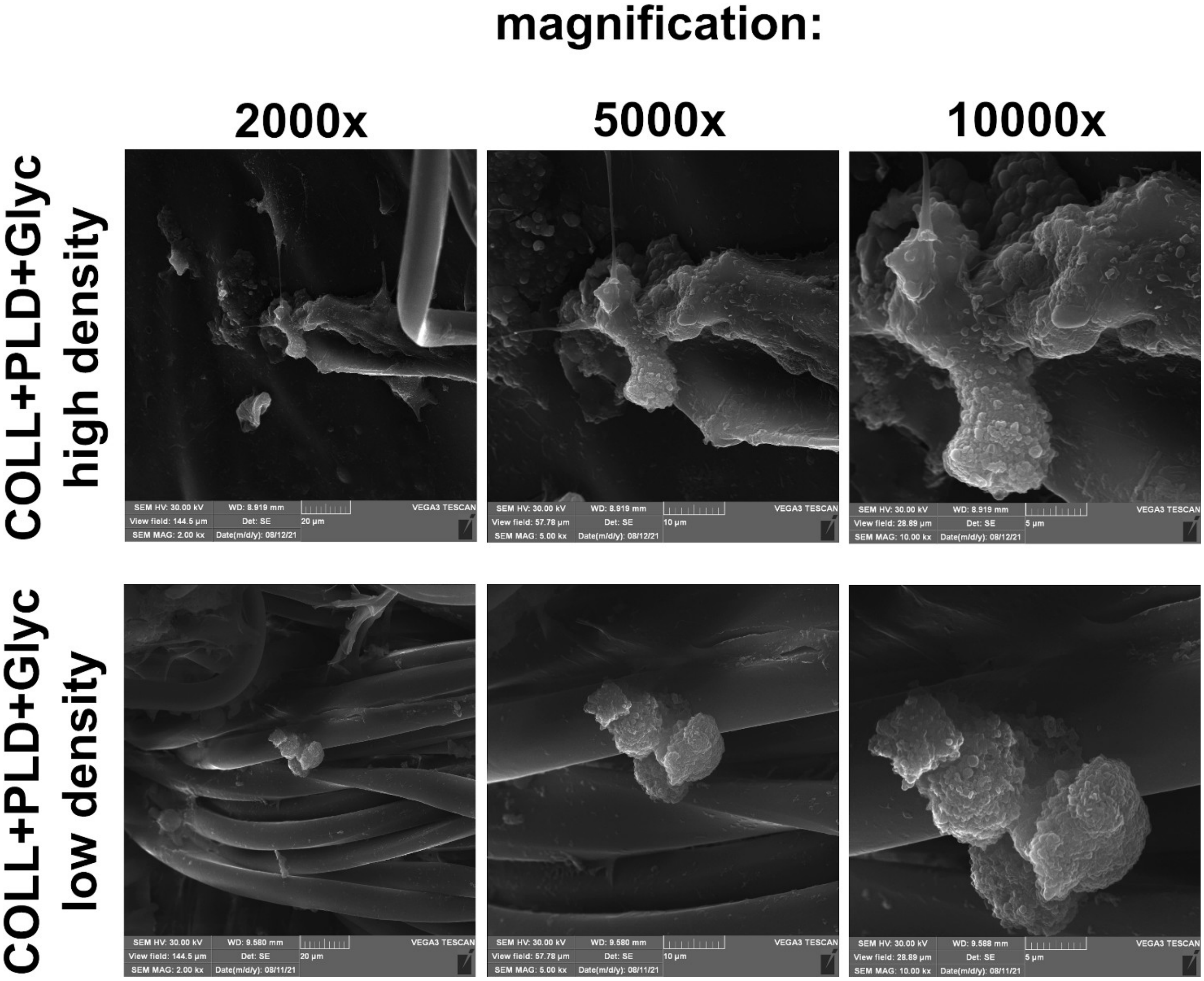

Figure S4. Uncropped and not rotated images of the HUVEC cells at sites with high and low density, grown on the Coll+PLD+Glyc prostheses for 4 days – SEM
